# Treatment Persistence and Variations in Prescribing Oral, Injectable, and Inhaled Corticosteroids: A Population‐Based Drug Utilisation Study

**DOI:** 10.1002/pds.70153

**Published:** 2025-04-30

**Authors:** Eleni Domzaridou, Matthew J. Carr, David M. Williams, Anthony J. Avery, Tjeerd van Staa, D. Aled Rees, Darren M. Ashcroft

**Affiliations:** ^1^ Nuffield Department of Population Health University of Oxford Oxford UK; ^2^ National Institute for Health and Care Research (NIHR) Greater Manchester Patient Safety Research Collaboration (GM PSRC), School of Health Sciences, Faculty of Biology, Medicine and Health University of Manchester Manchester UK; ^3^ Centre for Pharmacoepidemiology and Drug Safety, Division of Pharmacy & Optometry, School of Health Sciences, Faculty of Biology, Medicine and Health University of Manchester Manchester UK; ^4^ Neuroscience and Mental Health Innovation Institute Cardiff University Cardiff UK; ^5^ Centre for Primary Care, School of Medicine University of Nottingham Nottingham UK; ^6^ Centre for Health Informatics, Imaging and Data Science, Faculty of Biology, Medicine and Health University of Manchester, Manchester Academic Health Science Centre Manchester UK

**Keywords:** corticosteroids, treatment coverage, treatment persistence

## Abstract

**Purpose:**

To examine variation in oral, injectable, and inhaled corticosteroid (CS) prescribing in primary care, exploring treatment persistence and coverage.

**Methods:**

We examined patient‐level electronic health records from English general practices in the Clinical Practice Research Datalink Aurum database. We delineated a cohort of new users of oral, injectable, or inhaled CS with prescriptions issued between January 1, 2000, and June 30, 2021. Lorenz curves assessed potential prescribing skewness, and Kaplan–Meier (KM) plots estimated treatment persistence. The Proportion of Patients Covered (PPC) method estimated the proportion of patients still covered by treatment 1 year after initiation.

**Results:**

We observed 1 942 571 CS users across 1471 general practices, with 20% of oral and inhaled CS users accounting for almost 80% of total CS use. Older patients with comorbidities including respiratory diseases (13.5%), skin conditions (5.8%), or inflammatory bowel diseases (1.6%) were more likely to be prescribed higher doses. The KM plots showed that 20% of oral and 50% of inhaled CS users were persistent after one and 2 months, respectively. The PPC method indicated that 30% of oral and 60% of inhaled CS users were covered by treatment 6 months post‐initiation. Some variation was observed when different grace periods were applied. Combined use of oral and inhaled CS was observed for 6.9% of patients.

**Conclusion:**

A fifth of patients receiving CS accounted for over 80% of oral and inhaled CS prescribing in primary care. Identifying these patients is crucial for targeting future interventions to promote patient safety and cost‐effective CS use.

**Summary**

- Prescribing of oral and inhaled corticosteroids in primary care showed that 20% of users accounted for 80% of total prescriptions.
- Higher users of corticosteroids were older, with comorbidities including inflammatory bowel diseases, respiratory disease, or rheumatoid arthritis.
- Kaplan–Meier plots showed varying treatment persistence; the proportion of persistent patients after 1 month was 20% for oral corticosteroid users, and after 2 months was 50% for inhaled corticosteroid users.
- The proportion of patients covered by treatment 6 months post‐initiation was 30% for oral and over 60% for inhaled corticosteroids.

## 1 Introduction

Corticosteroids (CS) are used to treat multiple conditions such as allergies, eczema, asthma, chronic obstructive pulmonary disease (COPD), inflammatory diseases, and adrenal insufficiency, and have also been used widely as an effective treatment against severe Covid‐19 infection [1]. It has been estimated that between 1% and 3% of the general population use systemic (oral or injectable) CS [2, 3, 4], often prescribed as long‐term therapy [2]. In the UK, it has been estimated that 1.4% of patients over the age of 55 years received oral CS continuously for at least 3 months [5]. Furthermore, national drug utilisation data from the USA (1999–2008) and the UK (1989–2008) showed a rising incidence of long‐term CS prescriptions despite increased use of disease‐modifying drugs [2, 6].

CS are available through multiple administration routes, dosages, and treatment durations. Although they have been used widely, systemic CS may lead to several adverse events, including immunosuppression, osteoporosis, fractures, adrenal suppression, diabetes, psychiatric disturbances, dermatologic and cardiovascular events [7], as well as increased risk of hyperglycemia, especially for patients with diabetes [8]. It is important to study CS drug utilization and highlight variations in use to identify high users who are potentially at greatest risk of adverse events. Furthermore, understanding variation in prescribing and treatment adherence is crucial in this context, as inconsistent or prolonged CS use may further exacerbate the risk of adverse events.

Medication adherence is a concept that describes the degree to which a patient receives medications as advised. The EMERGE guidelines highlight three key phases: (a) treatment initiation; (b) implementation of the prescribed dosing; and (c) persistence with treatment [9, 10]. Nonadherence may occur at any stage, for instance, when patients do not initiate or delay treatment, take additional doses or omit doses, or if treatment is discontinued. Two of the methods that can measure treatment persistence are: (a) the refill‐gap method that measures persistence based on gaps between refills of prescriptions, and (b) the proportion of patients covered (PPC) method, which estimates the proportion of patients covered by treatment at a specific time after treatment initiation [11].

This study aimed to report variation in prescribing, treatment persistence and combined treatment patterns among patients who were prescribed oral, injectable and inhaled CS in primary care in England.

## 2 Methods

### 2.1 Data Sources

We examined anonymised, patient‐level electronic health records (EHRs) from general practices that contributed data to the Clinical Practice Research Datalink (CPRD) Aurum database [12]. The CPRD is one of the largest EHR databases in the world and has information pertaining to prescriptions, medical diagnoses, laboratory tests, referrals, immunisation, and demographic data [13]. The database covers almost 25% of the UK population and is representative in terms of age, gender, and ethnicity [12]. The study cohort was linked to practice‐level Index of Multiple Deprivation (IMD) quintiles. IMD data include information on relative deprivation and is measured across 32 844 small geographic areas or neighborhoods that include on average 1600 residents and are known as Lower‐level Super Output Areas (LSOAs) [14]. The IMD score is derived from variables that reflect variability in factors such as income, employment, education, health, crime, living environment, and access to services.

### 2.2 Study Design and Exposure Measures

New CS users, observed from 1st January 2000 until 30th June 2021, were included in the study cohort (Figure S1). Patients were included in the study cohort if they had at least 1 year of registration with their general practice and no history of CS exposure during this period, with data that were acceptable for research purposes and available linkage to IMD data (Figure S2). CS exposure was identified using prescription dates; patients with at least one prescription during their observation period were considered exposed. Product code lists of oral, injectable, or inhaled CS prescriptions were developed and reviewed by coinvestigators (ED and DMA). The first prescription issue date during their observation period was designated as the index date. Patients were followed up until the earliest occurrence of: (a) last date of data collection; (b) death; or (c) end of the study (30th June 2021), whichever came first.

Treatment duration was defined using prescription issue dates and duration data wherever available. The handling of missing data pertaining to oral treatment duration followed an established published protocol [15], according to which treatment duration was calculated based on individual‐level available data as the ratio of the total prescribed quantity over daily dose. In cases whereby either the quantity or daily dose data were missing, treatment duration was imputed by using the median duration among the same individual and the same product code prescribed. For patients who lacked duration data, the median duration of either another product code of the same drug substance was used or the median duration of the same product code of patients at the same age group or gender was applied. Duplicated prescriptions and overlaps were identified and added at the end of the last prescription if the strength of the prescribed substance was smaller than 10 mg, indicating a new prescription, and were ignored otherwise. This decision was informed by DAR and DMW and based on their clinical experience. For inhaled CS, treatment episodes of 30‐day duration were defined, and in cases where new prescriptions were issued within less than 60 days of the completion of the previous treatment episode, episodes were treated as continuous according to previously published studies [16, 17]. Therefore, all patients who were exposed to inhaled CS were assumed to be exposed for at least 30 days. Due to insufficient data on the duration of use for injectable products, the number of prescriptions issued within specific time frames was used instead of calculating the daily dose. READ and SNOMED code lists from primary care records were applied to identify comorbidities at baseline.

### 2.3 Data Analysis

Skewness in oral and inhaled CS prescribing was assessed using the Lorenz curve [18], whereby the y‐axis represents the cumulated percentile of CS prescribed (expressed as prednisolone and beclomethasone equivalents for oral and inhaled CS, respectively) and the x‐axis represents the percentile of people who were prescribed CS during the observation period.

Kaplan–Meier (KM) plots were used to estimate the proportion of patients who were treatment persistent from initiation until their first treatment break. The proportion of patients covered (PPC) method was used to estimate the proportion of patients who were covered by treatment at a specific timepoint following treatment initiation. Patients who are no longer covered by treatment are excluded from the numerator. Patients reentered the numerator if there was a new prescription recorded. For both the KM and PPC analyses, the follow‐up was restricted to 1 year after the index date because very few patients were exposed to CS for longer periods. A grace period of 30 days was applied to allow for late refills. Potential changes in the observed KM and PPC patterns were examined in sensitivity analyses by modifying the applied grace periods.

The prevalence of oral and inhaled CS use, stratified by gender, age groups, and dose (applied categories for oral: 0–2.5 mg, 2.5–7.5 mg, > 7.5 mg; inhaled: 0–500 μg, 500–1000 μg, > 1000 μg) [4, 19] was estimated by dividing the total treatment duration of oral or inhaled CS by the total follow‐up. KM survival analysis was applied to show the proportion of persistent patients, stratified by comorbidities at baseline.

In secondary analyses, combined treatment patterns were studied by examining overlapping episodes of oral and inhaled CS during the first year of every patient's follow‐up. All patients who were prescribed CS anytime within the observation period were included, and prescription rates were calculated by dividing the total number of patients who were prescribed CS by the corresponding person‐years of follow‐up. In sensitivity analyses, a rolling time window of audit dates was applied, representing the 30th of June 2005, 2010, 2015, and 2019, respectively, to examine potential changes in prescribing rates over time and check whether the results were sensitive to the choice of audit date.

All analyses were conducted using R version 4.4.1.

## 3 Results

We identified 1 942 571 patients of whom 1 375 099 were prescribed oral CS, 140078 injectable and 912 454 inhaled CS across 1471 GP practices in England. Across the total CPRD population (15 681 632 patients), 8.8% of patients were prescribed oral CS, 0.9% of injectable CS, and 5.8% inhaled CS. Prednisolone (91.5%), methylprednisolone (75.5%), and beclometasone (84.2%) were the most frequently prescribed oral, injectable, and inhaled CS, respectively. More than half of the patients (54.6%) were female, 67.6% were older than 35 years, and 24.1% were enrolled with GP practices in the most deprived residential quintiles of England. The median follow‐up was 6.8 years (interquartile range, IQR 2.8–12.5). Among patients receiving oral or inhaled CS, 67% were enrolled with GP practices in the 3rd, 4th, or 5th most deprived residential quintiles of England, with the corresponding number of patients receiving injectable CS being 57%. Regarding regional variation, nearly 33% of patients receiving either oral or inhaled steroids and 47% of patients in injectable CS were enrolled with GP practices in the South of England. Table 1 shows the baseline characteristics of the study cohort.

**TABLE 1:** Characteristics of all patients in the study cohort according to corticosteroid formulation.

	Oral	%	Injectable	%	Inhaled	%	*p*
Patients	1 375 099	—	140 078	—	912 454	—	—
Person‐years of follow‐up (median, interquartile range)	6.1 (2.4–11.3)	—	8.6 (3.9–12.6)	—	7.5 (3.3–13.4)	—	—
Gender = Male	616 447	(44.8)	59 733	(42.6)	421 422	(46.2)	< 0.001
Age group at index date							
0–18	177 510	(12.9)	2131	(1.5)	213 005	(23.3)	< 0.001
18–35	202 726	(14.7)	16 740	(12.0)	156 248	(17.1)	
35–45	204 540	(14.9)	27 393	(19.6)	134 487	(14.7)	
45–55	243 279	(17.7)	32 514	(23.2)	140 947	(15.4)	
55–65	248 646	(18.1)	30 339	(21.7)	131 508	(14.4)	
65–75	199 372	(14.5)	21 415	(15.3)	93 728	(10.3)	
> 75	99 026	(7.2)	9546	(6.8)	42 531	(4.7)	
Region							
East Midlands	33 133	(2.4)	3783	(2.7)	20 536	(2.3)	< 0.001
East of England	61 746	(4.5)	6457	(4.6)	43 280	(4.7)	
London	165 212	(12.0)	13 126	(9.4)	152 042	(16.7)	
North East	59 661	(4.3)	329	(0.2)	33 424	(3.7)	
North West	295 522	(21.5)	18 439	(13.2)	177 783	(19.5)	
South Central	157 540	(11.5)	23 989	(17.1)	111 138	(12.2)	
South East Coast	117 683	(8.6)	9422	(6.7)	77 284	(8.5)	
South West	181 876	(13.2)	32 162	(23.0)	114 025	(12.5)	
West Midlands	250 682	(18.2)	28 539	(20.4)	153 114	(16.8)	
Yorkshire & The Humber	51 350	(3.7)	3832	(2.7)	29 487	(3.2)	
Unknown	694	(0.1)	0	(0.0)	341	(< 0.1)	
Index of multiple deprivation							
1 (least deprived)	226 260	(16.5)	32 835	(23.4)	151 060	(16.6)	< 0.001
2	228 546	(16.6)	26 918	(19.2)	150 174	(16.5)	
3	283 195	(20.6)	23 492	(16.8)	185 670	(20.3)	
4	294 406	(21.4)	32 909	(23.5)	198 636	(21.8)	
5 (most deprived)	342 692	(24.9)	23 924	(17.1)	226 914	(24.9)	
Comorbidities at baseline							
Cardiovascular diseases							
Heart failure	12 759	(0.9)	1004	(0.7)	6841	(0.7)	< 0.001
Myocardial infarction	16 742	(1.2)	1789	(1.3)	8730	(1.0)	< 0.001
Angina	23 245	(1.7)	2586	(1.8)	12 138	(1.3)	< 0.001
Atrial fibrillation	18 968	(1.4)	1787	(1.3)	8444	(0.9)	
Respiratory system diseases							
Asthma	120 774	(8.8)	7336	(5.2)	122 499	(13.4)	< 0.001
Chronic obstructive pulmonary disease	40 878	(3.0)	1833	(1.3)	33 725	(3.7)	< 0.001
Cancer	64 409	(4.7)	5642	(4.0)	20 928	(2.3)	< 0.001
Multiple sclerosis	16 742	(1.2)	1789	(1.3)	8730	(1.0)	< 0.001

Comorbidities at baseline							
Inflammatory conditions							
Rheumatoid arthritis	9331	(0.7)	1118	(0.8)	3573	(0.4)	< 0.001
Systemic lupus erythematosus	856	(0.1)	70	(< 0.1)	371	(< 0.1)	< 0.001
Inflammatory bowel disease	24 067	(1.8)	1829	(1.3)	12 595	(1.4)	< 0.001
Osteoarthritis	44 264	(3.2)	9587	(6.8)	29 176	(3.2)	< 0.001
Diabetes							
Type 1	3050	(0.2)	520	(0.4)	1892	(0.2)	< 0.001
Type 2	33 040	(2.4)	4474	(3.2)	16 524	(1.8)	< 0.001
Chronic kidney disease							
Stage 3–5	27 505	(2.0)	2894	(2.0)	10 365	(1.1)	< 0.001
Chronic liver disease	6667	(0.5)	651	(0.5)	3427	(0.4)	< 0.001
Skin conditions							
Eczema	72 230	(5.3)	6265	(4.5)	52 913	(5.8)	< 0.001
Psoriasis	17 314	(1.3)	1947	(1.4)	9260	(1.0)	< 0.001
Rosacea	9167	(0.7)	1246	(0.9)	4683	(0.5)	< 0.001
Seborrheic dermatitis	6681	(0.5)	652	(0.5)	4088	(0.4)	< 0.001
Lichen planus	4458	(0.3)	524	(0.4)	1884	(0.2)	< 0.001
Pemphigus/pemphigoid	1087	(0.1)	29	(< 0.1)	146	(< 0.1)	< 0.001
Hidradenitis	925	(0.1)	82	(0.1)	521	(0.1)	0.009
Drug substance							
Prednisolone	1 258 729	(91.5)	924	(0.7)	—	—	—
Dexamethasone	127 403	(9.3)	1206	(0.9)	—	—	—
Beclometasone	—	—	—	—	768 157	(84.2)	—
Budesonide	—	—	—	—	160 785	(17.6)	—
Fluticasone	—	—	—	—	243 787	(26.7)	—
Methylprednisolone	—	—	105 738	(75.5)	—	—	—
Hydrocortisone	—	—	11 579	(8.3)	—	—	—
Triamcinolone	—	—	33 711	(24.1)	—	—	—

The median treatment duration among oral CS users was 28 days (IQR 28–36) and 30 days (IQR 30–133) among inhaled CS users. Only 2% received oral CS for longer than a year, with 80% of patients being on treatment for 44 days or fewer, while a small subset remained on treatment for significantly longer, with the maximum duration reaching 7542 days, which suggests a highly skewed distribution. Moreover, oral users had a median of 5 (IQR 2–14) treatment episodes, with the corresponding numbers for inhaled CS being 3 (IQR 1–6). Fifty‐three percent (74250) of injectable CS users received only one prescription during their follow‐up, with 20.6% (28818) receiving more than one prescription within 6 months, 11.8% (16456) within 6–12 months, 22.3% (31225) within 1–5 years, and 15.3% (21461) more than 5 years since the first prescription was issued. In terms of the number of prescriptions, 39.6% (55437) patients received between two and five, and 6.5% (9056) patients had more than five prescriptions of injectable CS.

**FIGURE 1:**
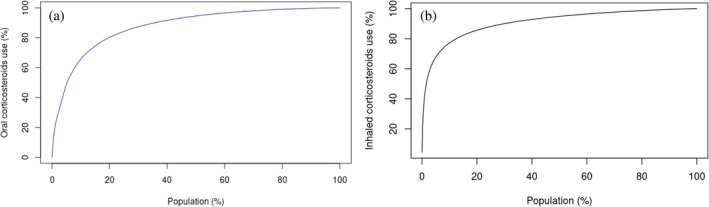
Lorenz‐curves showing the percentile of patients who were prescribed oral or inhaled corticosteroids versus the cumulated percentile of corticosteroid use expressed as prednisolone and beclometasone equivalents, respectively.

**FIGURE 2:**
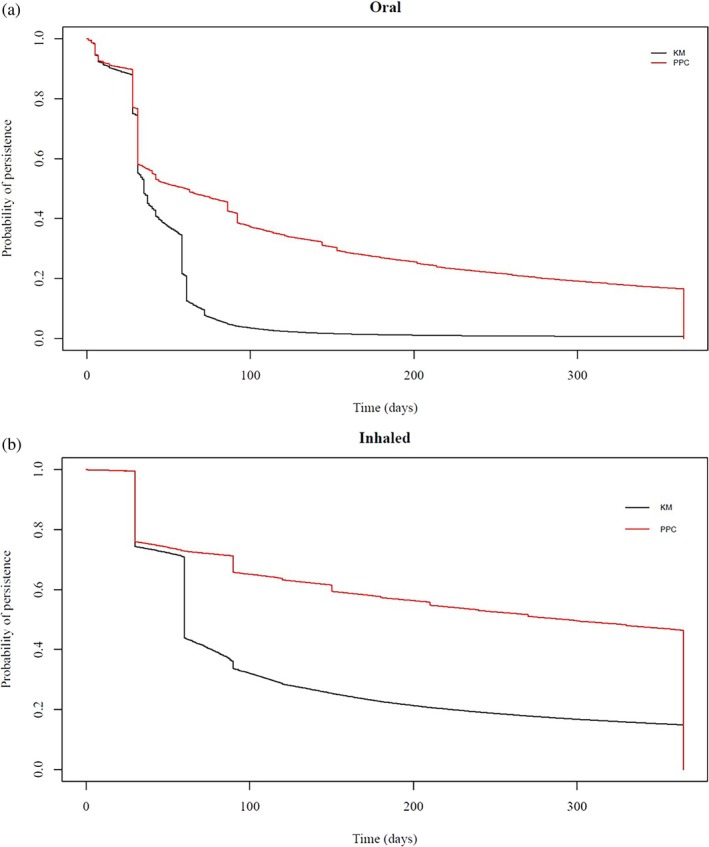
Treatment persistence patterns using Kaplan–Meier (KM) plots, and the Proportion of Patients covered (PPC) method among oral and inhaled corticosteroid users during their first year of follow‐up.

The Lorenz curve in Figure 1 demonstrates that nearly 20% of oral and inhaled users accounted for 80% of CS use. A comparison between KM and PPC plots for oral and inhaled CS in Figure 2 shows distinct patterns across the two formulations. Almost 80% of oral CS users received continuous treatment during the first month of their follow‐up, with a sharp drop to 20% (Hazard Ratio, HR: 0.205; 95% Confidence Interval, CI: 0.200–0.206) after 2 months. The proportion of persistent users for longer than 100 days was less than 3% (HR: 0.026; 95% CI: 0.026–0.026). Similar patterns were observed for inhaled formulations, with approximately 50% (HR: 0.475; 95% CI: 0.471–0.477) of persistent users 2 months following treatment initiation. The PPC method showed that almost 30% (HR: 0.301; 95% CI: 0.300–0.304) of oral and 60% (HR: 0.600; 0.600–0.603) of inhaled users were covered by treatment 6 months following treatment initiation. In sensitivity analyses, the KM plots showed distinct patterns for oral and inhaled CS when different grace periods of 30, 60, and 90 days were applied (Figure S3). Similarly, in Figure S4, the PPC method showed that most patients were covered by treatment 6 months following treatment initiation, with the application of different grace periods shifting the observed patterns proportionately.

**FIGURE 3:**
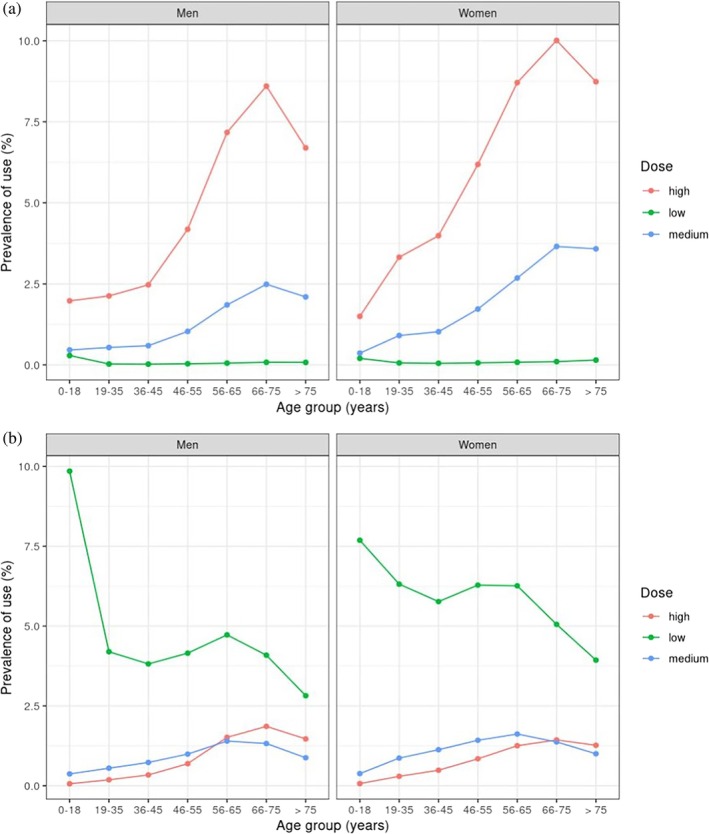
Use of oral and inhaled corticosteroids, stratified by gender, age group, and dose (dose defined as high (> 7.5 mg); medium (2.5–7.5 mg); low (0–2.5 mg) for oral, and as high (> 1000 μg); medium (500–1000 μg); low (0–500 μg) for inhaled formulations).

**FIGURE 4:**
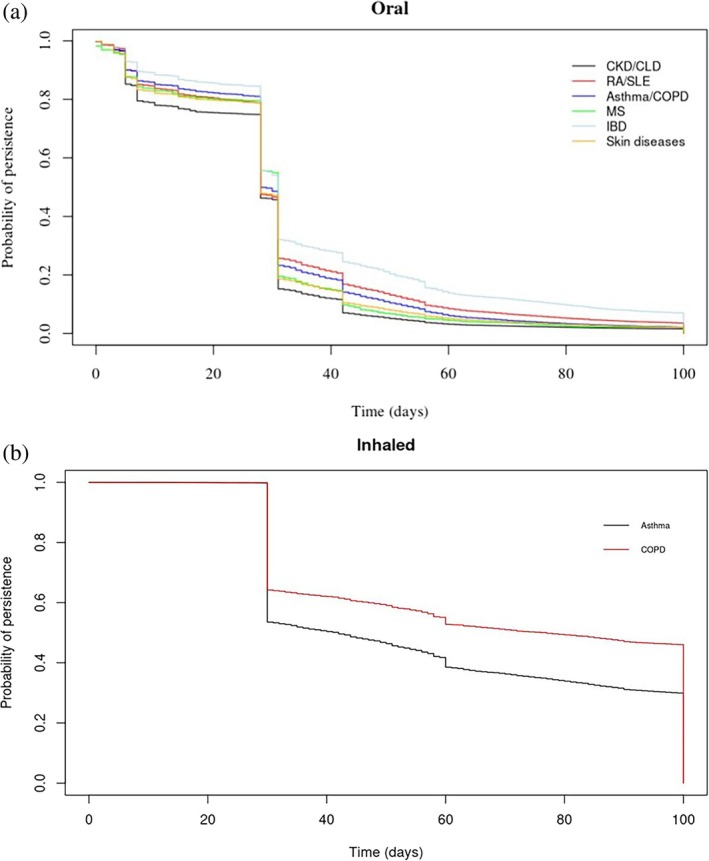
Treatment persistence patterns using Kaplan–Meier plots among oral and inhaled corticosteroid users during their first 100 days of follow‐up, stratified by comorbid conditions at baseline. Skin diseases included eczema, lichen planus, pemphigus, pemphigoid, and hidradenitis suppurativa. CKD, Chronic kidney disease; CLD, Chronic liver disease; COPD, Chronic obstructive pulmonary disease; IBD, Inflammatory bowel disease; MS, Multiple sclerosis; RA, Rheumatoid arthritis; SLE, Systemic lupus erythematosus.

Examination of the dose impact on CS prevalence (Figure 3) showed that a higher proportion of women older than 35 years and men older than 45 years were using high daily doses of oral CS (> 7.5 mg), while patients on low doses had the lowest prevalence of use. In contrast, 74.6% of patients on inhaled CS were using low daily doses (< 500 μg), followed by medium (13.9%) and high doses (11.5%), with some overlap among older patients. Post hoc analysis regarding asthma and COPD CS users showed that most asthma patients were prescribed low doses, whilst COPD patients older than 45 years were prescribed high or medium doses (Figure S5). Stratification of the KM plots according to comorbidities at baseline (Figure 4) showed that 50% (HR: 0.50; CI: 0.51–0.55) of all patients with the examined comorbidities were most likely to remain on continuous treatment after 28 days. Post hoc analyses indicated that the results were sensitive to changes in treatment duration, suggesting that longer treatment episodes may increase the probability of persistence (Figure S6). Among inhaled users, patients with COPD remained on continuous treatment for longer periods compared to patients with asthma.

Finally, examination of combined treatment patterns, including patients who were prescribed oral and inhaled CS, showed that most patients were prescribed solely oral formulations, with 6.9% (133524) of the studied population receiving concurrently oral and inhaled CS. Sensitivity analyses using June 30th as an audit date between 2005 and 2021 showed slightly different prescription rates over the study period with no impact on the overall inferences (Table S1).

## 4 Discussion

We found that 20% of users of oral and inhaled CS contributed 80% of the total prescribing in general practice. In addition, KM plots showed that 50% of patients who were prescribed inhaled formulations were treatment persistent for 2 months until their first treatment gap. Almost 20% of oral users were treatment persistent for a month. The PPC method showed that less than 20% of oral and 50% of inhaled CS users were covered by treatment during the first year of follow‐up. We also observed that the prevalence of high‐dose oral CS use increased significantly with age, peaking in the 66–75 age group for both men and women. Medium dose also rose with age but to a lesser extent, while low‐dose use remained consistently low across all age groups, with the increase in prevalence for high and medium doses being more pronounced in women compared to men. Finally, patients with inflammatory bowel disease, rheumatoid arthritis, systemic lupus erythematosus, and respiratory conditions were most likely to remain on continuous oral CS treatment, while asthma patients on inhaled CS had shorter treatment periods compared to COPD patients.

Multiple methods allow the examination of treatment persistence using EHRs [20]. For instance, treatment persistence can be assessed using KM plots and the PPC method, although only the PPC method is often applied, whilst skewness in data dosage can be visually illustrated using Lorenz curves [20]. A previous study that examined skewness of CS exposure and focused on oral formulations for treating asthma found that 10% of patients was accounting for more than 50% of dispensed CS [21]. Systematic reviews of the methods used to assess adherence using EHR databases indicate a lack of clear consensus regarding the methodology, concluding that, ideally, both treatment duration and implementation should be considered. This is because in the PPC method, patients with longer treatment gaps that might indicate low implementation are excluded [22, 23]. This means that patients can cycle in and out of the numerator as many times as they are prescribed CS. In contrast, using the KM plots, patients with longer treatment gaps are censored. This allows patients with low implementation to be included in the analysis, which may influence the observed patterns in the PPC curves. Therefore, the extent to which the observed patterns that include patients with low implementation are representative depends on the representation of those patients at the population level. Finally, in our study, changes in grace periods showed some variation, and this is important to consider, especially when the observation period is shorter than a year. The results from the PPC method were less sensitive to changes in grace periods compared to the KM plots.

In this study, we also observed that the use of high and medium oral CS doses increases with age. A previous study that examined the use of oral CS in the UK found that the highest use was by patients between 70 and 79 years, and patients with arthropathies were most likely to receive uninterrupted CS treatment [4]. Our findings generally align, but some key factors that might be responsible for the differences in treatment continuation according to comorbidities include the study's observation period, the population and sample size, differences in person‐years of follow‐up, and the definition of treatment episodes, or grouping of disease categories [2, 24]. In the sensitivity analysis for patients prescribed both oral and inhaled CS over time, crude rates appear to be declining, likely due to the increased availability and use of biologics for inflammatory conditions. However, our findings showed that 20% of users were associated with the highest prescribing, which emphasizes the importance of identifying these patients and supporting interventions to minimize potential risks in the future.

Although duration data for oral formulations were imputed using a published algorithm [15], and duration data pertaining to inhaled CS were determined based on the published literature [17], some degree of exposure misclassification is possible. For example, standard metered‐dose inhalers, such as beclomethasone, typically contain 200 doses. The maximum recommended dose for most CS inhalers is two inhalations twice daily, equating to four inhalations per day. Under conditions of full implementation, this regimen would result in an inhaler lasting approximately 50 days for most patients. Conversely, some patients may implement a lower dosing regimen, such as one inhalation twice daily, in which case an inhaler would last up to 100 days, which is the case for more than 30% of the patients, as the KM plot shows. Furthermore, in clinical practice, the majority of oral CS courses are prescribed for individuals experiencing exacerbations of asthma or COPD. Typical regimens for acute exacerbations include 6 to 8 tablets of 5 mg prednisolone daily for 5 to 7 days, resulting in a total course of 30 to 40 tablets. Similarly, another common regimen consists of 8 tablets of 5 mg prednisolone taken once daily for 5 days. A 30‐day treatment duration was assumed to delineate episodes of inhaled CS according to the literature, with a 30‐day grace period applied to capture late refills. Other published studies have utilised 60‐ or 90‐day episodes [25], however, we believe that this matter should be examined in sensitivity analyses. Additionally, the methods that were applied to handle duplicated and overlapping prescriptions may also have contributed, to some degree, to exposure misclassification. A previously conducted cross‐sectional study by Joseph et al. [26] utilised oral and injectable CS prescriptions from 78 patients and compared self‐reported CS use to prescription‐based CS exposure using the CPRD. The study found that 84.2% of patients receiving oral CS were correctly classified as being exposed (‘on treatment’) to CS and 87.5% were correctly classified as unexposed (‘off treatment’), whilst the concordance was low for patients receiving injectable CS, indicating that EHRs per se are not a reliable source to utilise exposure to injections [26]. This was the reason why our approach regarding injections was purely descriptive. The investigators also examined the impact that exposure has had on the relative risk of a hypothetical population and found that exposure misclassification may underestimate the risk for studied outcomes [26].

We acknowledge some potential limitations. For instance, data on medication supply were not available because there is no linkage between prescribed and dispensing data, nor were medication adherence data available. Furthermore, since adherence data were not available, we were unable to examine whether the medications were consumed as prescribed. This is a common limitation among studies that utilize EHRs. Moreover, the index date was assumed to represent treatment initiation, which might not necessarily indicate the exact date that patients initiated treatment; however, we accounted for this potential limitation by including patients who had at least 1 year of continuous registration with their general practice.

Another possible limitation is that patients with chronic conditions, severe diseases, or multimorbidity that need complex therapeutic schemes might have been exposed to CS for longer periods compared to patients who were prescribed CS occasionally (e.g., skin or lung infections). This is expected to influence the observed treatment persistence and/or combined treatment patterns, with patients treated for longer periods being those who have chronic medical conditions. A study demonstrated that users of oral CS with arthropathies remained on treatment for longer periods compared to patients with COPD [4]. Patterns of combined treatment that involved drugs other than CS have not been considered, nor were the reasons that might have led to early treatment discontinuation or switching to therapeutic alternatives (e.g., due to adverse events) investigated. Topical CS and other formulations used as short‐term therapies including intranasal, ocular, ear drops, or rectal CS were not examined due to recognised difficulties in determining treatment duration [27]. Future research should consider whether the observed treatment persistence patterns insist for patients with multiple chronic conditions and those who are prescribed complex therapeutic schemes.

In conclusion, we found that a fifth of patients receiving CS accounted for over 80% of oral and inhaled CS prescribing in primary care. Those patients are more likely to be older, with comorbidities including inflammatory bowel diseases, respiratory disease, or rheumatoid arthritis and systemic lupus erythematosus. Identification of those patients will be important for targeting future interventions to promote patient safety in the use of CS in a cost‐effective manner.

### 4.1 Plain English Summary

We studied how corticosteroids (CS) are prescribed in general practices across England. We identified 1 942 571 new users of oral, injectable, or inhaled CS from 1471 general practices. To visualize differences in prescribing patterns, we used Lorenz curves. We also used Kaplan–Meier (KM) plots to examine how long patients continued their treatment. The Proportion of Patients Covered (PPC) method was employed to estimate how many patients were still receiving treatment 1 year after starting. We observed variations in prescribing oral and inhaled CS, with 20% of patients accounting for nearly 80% of all CS use. The KM plots indicated that about 20% of oral and 50% of inhaled CS users remained on treatment after 1 and 2 months, respectively. The PPC method showed that 30% of oral and 60% of inhaled users were covered by treatment 6 months after initiation. Additionally, 6.9% of patients received both oral and inhaled CS at the same time. We observed a subgroup of frequent users, typically older patients with chronic conditions like respiratory disease, which may inform future patient safety interventions related to CS treatment.

## Author Contributions

D.M.A. conceptualised the study. E.D., D.M.A., and M.J.C. contributed to study design. E.D. developed the dataset and analysed the data. E.D. and M.J.C had full access to and verified the underlying study data. E.D. drafted the manuscript. All authors contributed to the interpretation of data. All authors reviewed results, revised the manuscript, approved its final version, and agreed to be accountable for all aspects of the work.

## Ethics Statement

The study was approved by the CPRD Independent Scientific Advisory Committee (reference: 22_001811). No further ethical approval was needed.

## Conflicts of Interest

A.J.A. is the National Clinical Director for Prescribing for NHS England. The other authors declare no conflicts of interest.

## Supporting information


**Data S1.** Supporting Information.
