# Supplementary material for: Treatment Persistence and Variations in Prescribing Oral, Injectable, and Inhaled Corticosteroids: A Population‐Based Drug Utilisation Study
Source: Pharmacoepidemiol Drug Saf. 2025 Apr 30;34(5):e70153. doi: 10.1002/pds.70153 (PMC12042156; doi:10.1002/pds.70153)
Supplement: Supplementary file 1 — Data S1. Supporting Information. [file PDS-34-e70153-s001.docx]

**APPENDIX**

**Figure S1**: Study design

**
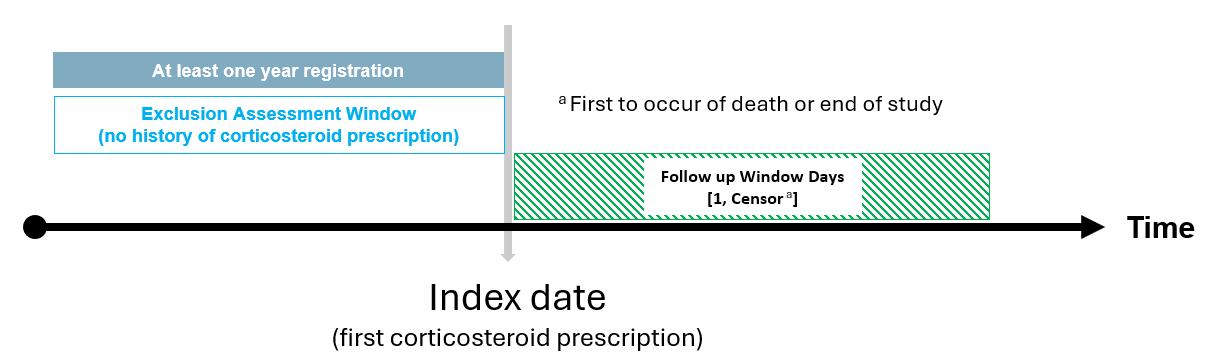
**

**Figure S2**: Data extraction flowchart.


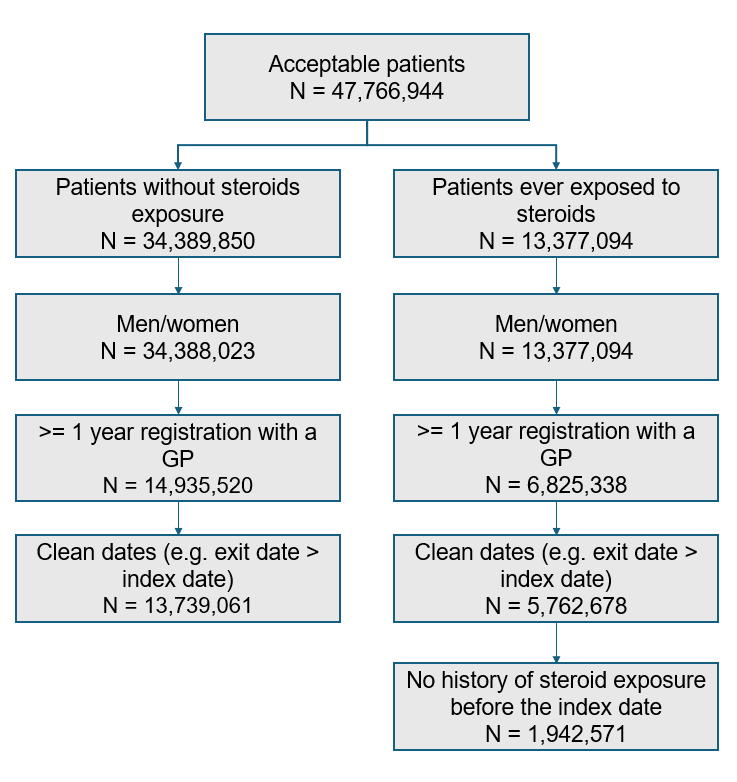


**Figure S3**: Treatment persistence patterns using Kaplan-Meier plots among oral and inhaled corticosteroid users during their first year of follow-up by applying different grace periods.


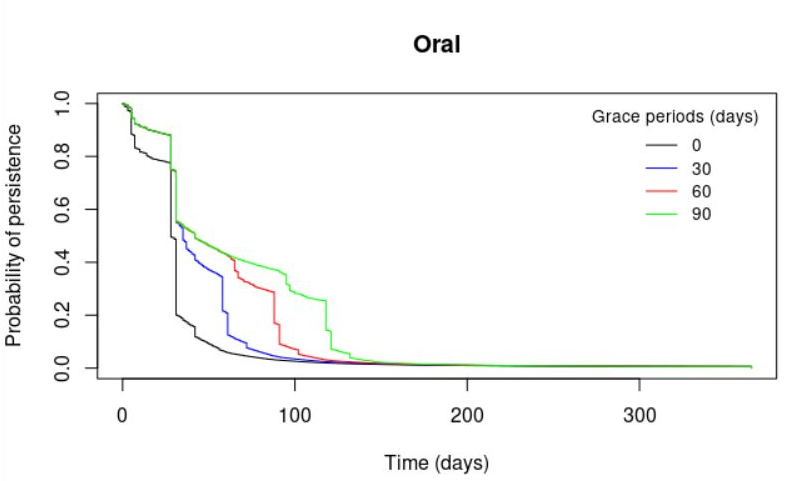


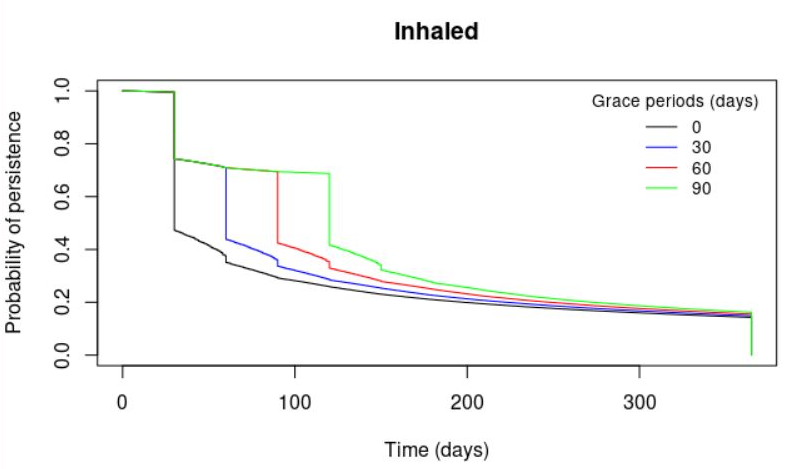


**Figure S4**: Treatment persistence patterns using the Proportion of Patients Covered (PPC) method among oral and inhaled corticosteroid users that remained on treatment during their first year of follow-up by applying different grace periods.


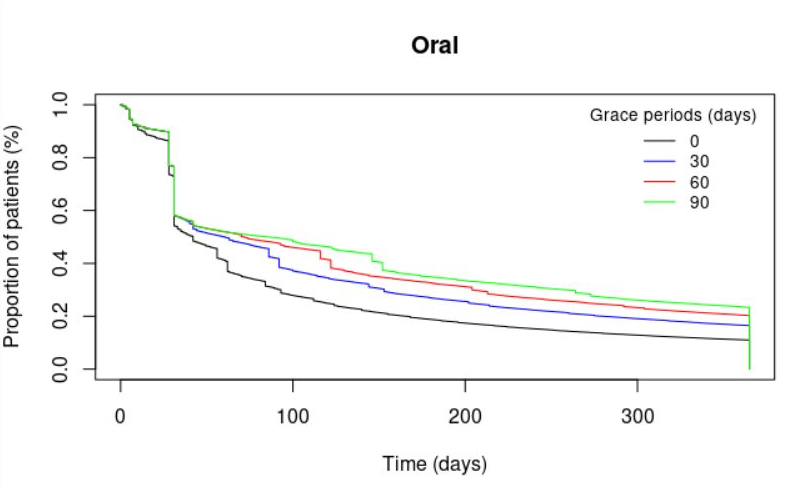


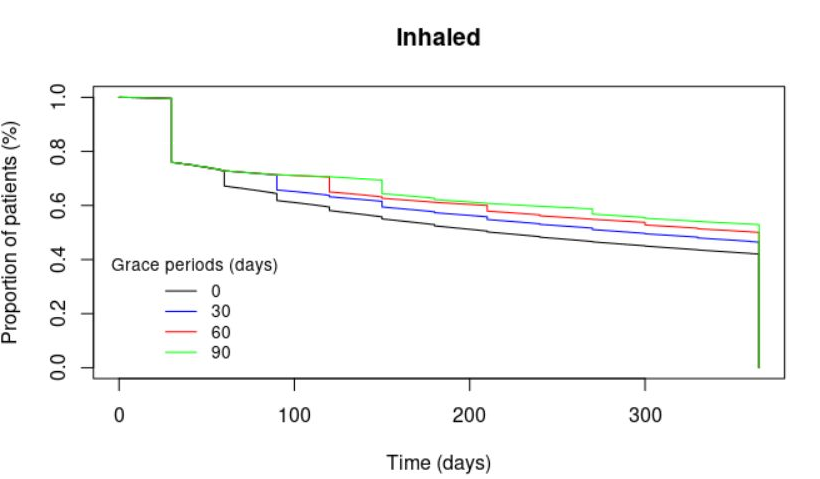


**Figure S5**: Use of inhaled corticosteroids among patients with asthma or chronic obstructive pulmonary disease (COPD), stratified by gender, age group, and treatment dose.

Asthma


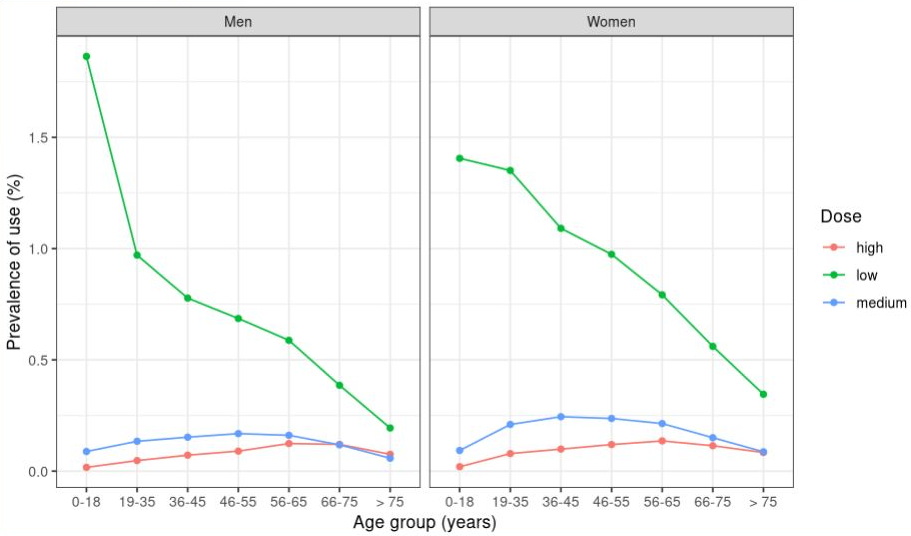


COPD


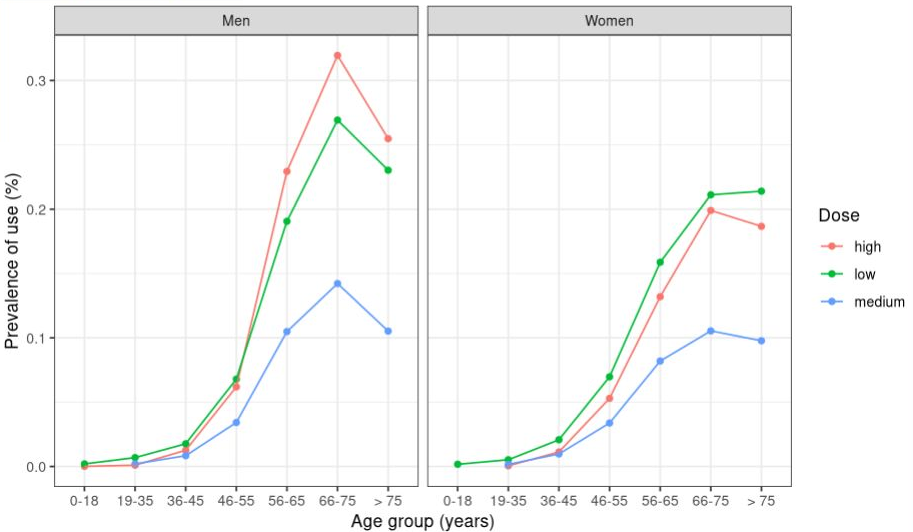


**Figure S6**: Sensitivity analyses included Kaplan-Meier plots among oral corticosteroid users during their first 100 days of follow-up, stratified by comorbid conditions at baseline, with treatment episodes of A – 14, B – 28, and C – 56 days.

A


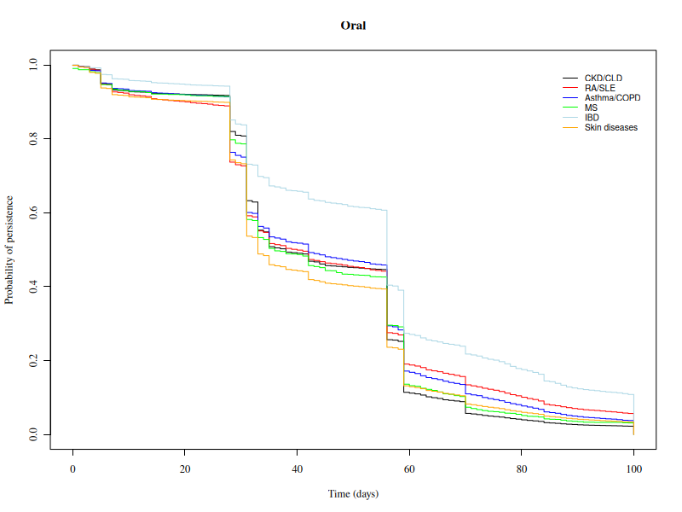


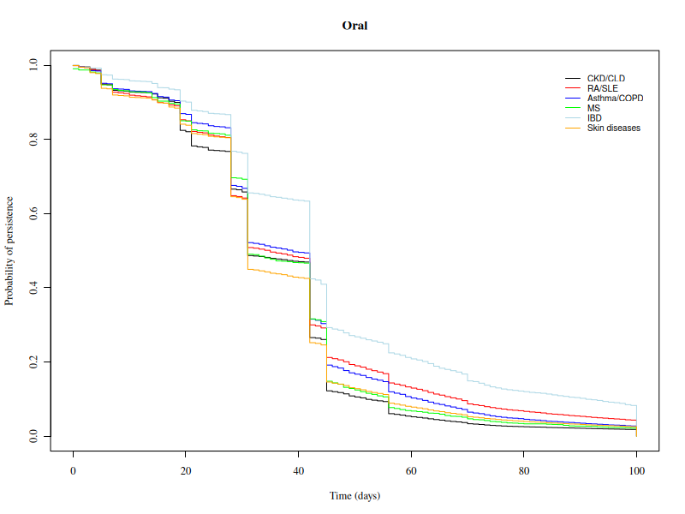
B

C
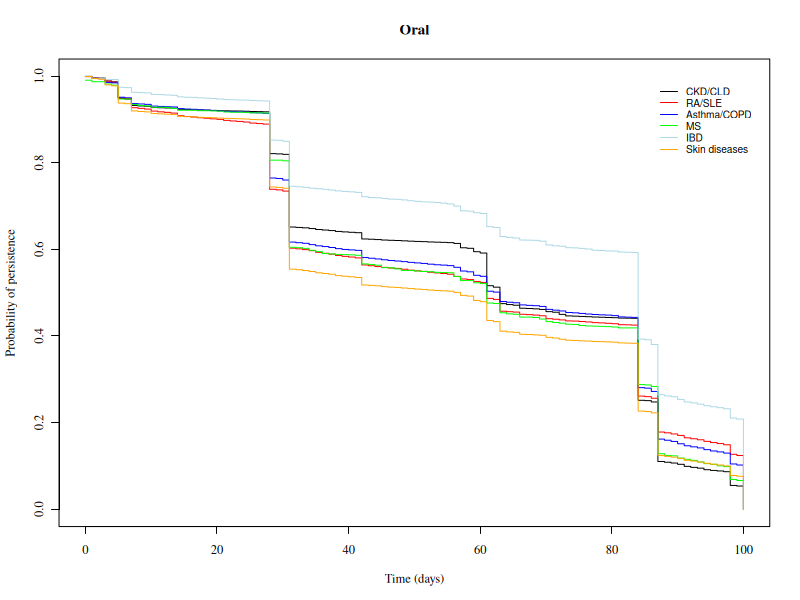


**Table S1**: Combined treatment patterns – sensitivity analysis

|  | **2000-2021** | | | **2005-2021** | | | **2010-2021** | | | **2015-2021** | | | **2019-2021** | | |
| --- | --- | --- | --- | --- | --- | --- | --- | --- | --- | --- | --- | --- | --- | --- | --- |
| **Mode** | **Patients*** | **PYE** | **CR** | **Patients** | **PYE** | **CR** | **Patients** | **PYE** | **CR** | **Patients** | **PYE** | **CR** | **Patients** | **PYE** | **CR** |
| Oral | 1,008,697 | 498,979 | 2.02 | 840,128 | 438,891 | 1.91 | 648,617 | 353,854 | 1.83 | 399,054 | 241,395 | 1.65 | 157,183 | 128,985 | 1.22 |
| Inhaled | 629,875 | 265,130 | 2.38 | 523,871 | 261,178 | 2.01 | 408,369 | 229,419 | 1.78 | 291,436 | 184,239 | 1.58 | 188,534 | 135,256 | 1.39 |
| Oral and inhaled | 133,524 | 107,381 | 1.24 | 121,017 | 85,059 | 1.42 | 108,244 | 84,884 | 1.28 | 87,450 | 77,332 | 1.13 | 52,483 | 51,693 | 1.02 |

*The difference between the total number of patients included in the cohort and the sum of the numbers in the second column of Table S1 arises due to patients who were prescribed combinations of oral or inhaled formulations, but not concurrently.

PYE: Person-years of corticosteroids exposure.

CR: Crude rate, calculated by dividing the number of patients with the number of person-years.
